# Current Potential Outcomes of Buccal Mucosal Graft Anterior Urethroplasty for Male Urethral Stricture: A Single-Centre Study in Nepal

**DOI:** 10.7759/cureus.70379

**Published:** 2024-09-28

**Authors:** Radheshyam Gupta, Honglei Wang, Suman Gupta, Wenxin An, Tao Xu, Nand Lal, Javed Iqbal, Chitaranjan Shah

**Affiliations:** 1 Urology Surgery, Vayodha Hospital, Kathmandu, NPL; 2 Urology Surgery, Nepal Korean Friendship Municipality Hospital, Kathmandu, NPL; 3 Urology Surgery, Harbin Medical University Cancer Hospital, Harbin, CHN; 4 Dental Surgery, National Medical College, Birgunj, NPL; 5 Physiology, School of Biomedical Sciences, Harbin Medical University, Harbin, CHN; 6 Physiotherapy, Dow University of Health Sciences, Karachi, PAK; 7 Urology, National Academy of Medical Sciences, Bir Hospital, Kathmandu, NPL

**Keywords:** anterior urethral stricture, balanitis xerotica obliterans (bxo), buccal mucosal graft (bmg), penile stricture, urethroplasty

## Abstract

Objective

This study evaluates the efficacy and potential complications of Buccal Mucosal Graft (BMG) urethroplasty for anterior urethral stricture over a 48-month follow-up.

Method

A retrospective review was conducted on 130 patients who underwent various types of BMG urethroplasty between 2012 and 2019. Data on patient demographics, stricture etiology, and anatomic site were collected. Adverse outcomes such as complications like erectile dysfunction (ED) persisting for over 12 months, and post-micturition dribbling (PMD) were analyzed to determine success rates, recurrent stricture risk factors, complications, and the definition of failure as stricture recurrence during the 48-month follow-up period.

Results

Of the 130 patients, there was a recurrence in 15.4 % (20 males), yielding a success rate of 84.6% (n=110). ED was reported in 11% (n=14) and PMD in 14% (n=18). All instances of ED were non-organic, and patients were administered oral phosphodiesterase type 5 (PDE5) inhibitors. These complications were observed in 20 patients (15.4%); with urinary fistula (3.0%), graft contracture (2.3%), graft failure (3.8%), urinary tract infection (UTI) (3.0%), and wound infection (2.3%) being the most prevalent after penile urethroplasty. Univariate analysis indicated age (31-50 years, >50 = P<0.05) at surgery, etiology (Balanitis Xerotica Obliterans (BXO)* *= P<0.05), stricture length (4.1-8 cm, >8 cm = P<0.05), and location as significant predictors of stricture recurrence. However, multivariate analysis highlighted penile location (P<0.05) as the sole independent predictor for restricture during the follow-up period.

Conclusion

BMG urethroplasty demonstrates a substantial 84.6% success rate in treating anterior urethral stricture over a 48-month follow-up period. This outcome underscores the advancements in healthcare quality in resource-limited settings in countries like Nepal.

## Introduction

A medical disorder called urethral stricture disease affected 300 men out of every 100,000 in 1998. Many treatment techniques have* *been recorded, including direct visual internal urethrotomy (DVIU) and anastomotic or augmentation urethroplasty using flaps and grafts [1]. Barbagli first described it in 1996 the dorsal onlay-free graft urethroplasty has been successfully used to treat penile and bulbar urethral strictures [2-3]*. *In the care of bulbar and penile urethral strictures that are not susceptible to excision and anastomosis, the use of a buccal mucosal graft (BMG) as a replacement for the urethral mucosa has become a well-established method. BMG urethroplasty has been performed using a variety of configurations, including dorsal onlay, dorsal inlay, and ventral onlay via a ventral sagittal urethrotomy approach, dorsolateral onlay with one-sided urethra mobilization, combined dorsal plus ventral double mucosal grafts, two-stage repairs, and augmented anastomotic urethroplasty [4-5]. The BMG has been widely used for urethroplasty and offers the advantages of being hairless and simple to harvest. The graft contains the whole thickness of the mucosal graft.

In Nepal, urological patients are grappling with a significant challenge due to urethral stricture, a difficult condition that affects the urethra. Only a few (three to four) center hospitals in Nepal have implemented BMG urethroplasty for the treatment of anterior urethral strictures, and this would affect the patients' long-term results. Young surgeons and healthcare practitioners' education and experience will also be altered because of this. Consequently, based on our information, the first scientific study on BMG urethroplasty in Nepal is reported here. Regardless of the stricture site and duration, it was first treated with regular DVIU and serial dilatation, which had unsatisfactory results.

This study's objectives are to describe the successful management of anterior urethral stricture and to pinpoint the risk factors for complications and stricture recurrence in all patients who have had buccal mucosal graft (BMG) urethroplasty at Vayodha Hospital, Kathmandu, Nepal, an ISO-certified facility.

This article was previously published as a preprint on the Research Square preprint server on 3rd July 2023 (https://www.researchsquare.com/article/rs-3045597/v1).

## Materials and methods

To evaluate the effectiveness of treating anterior urethral stricture with buccal mucosal graft urethroplasty, we conducted a retrospective hospital-based study. The study's retrospective data was gathered in the same location from January 2012 to January 2019. The Ethical Instructional Review Committee of Vayodha Hospital Pvt. Ltd issued approval VHPL-IRC/389/076/077.

The inclusion criteria for this research were male patients over the age of 12 who had been diagnosed with penile and bulbar anterior urethral strictures.

Sixty-eight individuals overall had bulbar strictures and 62 had penile and peno-bulbar urethral strictures. While 99 patients (76.1%) disclosed a history of recurrent stricture after DVIU, urethral dilatation, and urethroplasty in the past. A single stage of dorsal onlay was performed on 68 patients. Out of 68 patients having bulbar strictures, two patients recorded onlay bulbar urethroplasty, which was completed with a 6-month interval between the first and second phases. In Barbagli et al. [2], the dorsal onlay approach was used, which involved performing a dorsal stricturotomy into healthy tissue on each side of the stricture and then performing a dorsal onlay-free BMG urethroplasty. The 62 patients of penile and peno-bulbar underwent treatment fully opened by ventral midline penile incision. Out of the 62, only one case was performed during the second stage 6 months after the first stage - the same as described after penile urethroplasty.

Following preoperative outpatient clinic visits, preoperative history, clinical examination flow rate, post-voiding residual, and anterograde/retrograde urethrography, all patients were diagnosed with anterior urethral stricture. General and spinal anaesthesia were used throughout the procedure, along with nasal intubation for the bilateral cheek grafts and local anaesthesia up to the moment of graft harvesting. The same patients had a submucosal injection of a 2% xylocaine/200:000 adrenaline/saline mixture for the BMG harvesting procedure. Once the parotid duct has been identified, the borders of the graft are marked using a sterilised permanent marker pen. The bilateral cheek BMG was harvested with a typical 2.5 cm width and large strictures. The size of the graft was proportionate to the stricture length. A compressive gauze was kept in place and removed in the recovery region following precise hemostasis with bipolar electrocautery. All patients were instructed to use benzydamine hydrochloride-based mouthwash three times per day for a week following surgery.

A lithotomy posture was used for the patients. To assess a location, total or partial obstruction, and the length of the stricture, urethroscopy was performed using a semi-rigid ureteroscope (URS) with a size 6.5/7.5 FR tip. If a guidewire could not pass through the obstruction, it was considered to be completely blocked. If a URS could pass it, it was considered partial and might then be supplemented. However, in penile stricture, the penile urethra often invaded below the scrotum. As the guidewire was being induced, a longitudinal incision was created along the sub-scrotal raphe, and the bulbar urethral was examined [1]. The bulbospongiosus muscle was split along the middle. The corpus spongiosum was mobilized and detached from the corpus cavernosum. The urethra was opened dorsally by two reconstructive urologists and the part of the corpus cavernosum connected to the corpus spongiosum under direct vision and the BMG collected the graft visible. The harvested graft was then continuously sutured to the corpus spongiosum, corpus cavernosum, and urethra bilaterally using 4/0 Vicryl (cutting body with batch number 2443) and an appropriately sized silicon catheter. Finally, hemostasis was maintained without the need for a drain while the wound healed in layers.

**Figure 1:**
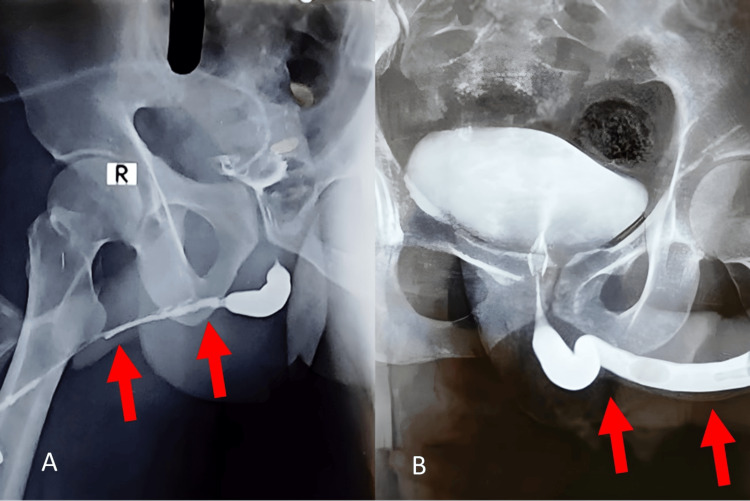
A. Urethrogram results shown pre-operative appearance of bulbar and penile urethral stricture, B. urethrogram post-operatively showing the augmented normal urethral site.

**Figure 2:**
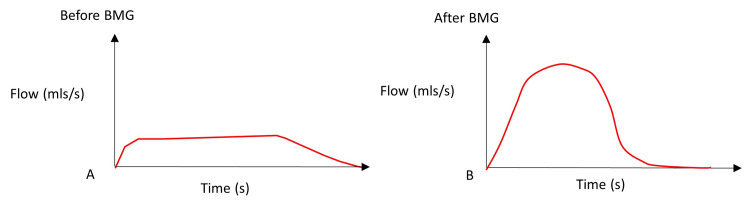
BMG Results Uroflowmetry: A. Before surgery results (Qmax) was 5.9 ml/s and B. Post-surgery follow-up result Qmax was 25 ml/s. BMG: buccal mucosal graft

On the operative day, the patient was in the Post Operative Room after surgery, and the patient was stable on the second day, they were moved to the general ward. Fusidic acid ointment was used while the gauze covering the perineal incision was removed for an alternative day dressing till the suture was out. On the first day, internal packing with gauze was completed at the BMG harvest location. Sips of water or non-spicy, cool oral fluid were provided on the second day, and on the third day, a soft diet was administered. Between the third to the fifth postoperative day, the patient was released. Third-generation cephalosporin or fluoroquinolones were administered intravenously while the patient was in the hospital, along with oral metronidazole and the same antibiotics intravenously for a further 14 days following discharge. When the routed ascending/descending urethrogram was done on all patients at 4 weeks, then from 3 up to 48 months after surgery (Figure 1), as well as post-second stage in cases of penile urethroplasty up to 36 months later, the urethral catheter was removed based on the lack of extravasation. Recently, a suprapubic catheter was placed as per standard, followed by the removal of the urethral catheter and a 3- to 7-day period of tightening. Upon successful urination, the suprapubic catheter was removed. The mean maximum flow rate (Qmax) of the preoperative uroflowmetry investigation was 5.9 (range 4.5-9) ml/s. 48 months after surgery, the mean Qmax was 25 (range: 12-30) ml/s during the postoperative follow-up period (Figure 2).

Data were gathered with follow-up from patient files and hospital records. Success and failure in terms of stricture recurrence, patient characteristics, the cause and anatomy of strictures, and the unfavourable results. Univariate statistical analysis was performed using t-test and chi-squared; multivariate analysis was done using regression analysis with P<0.05 indicating statistical significance using SPSS V.21.0 (IBM Corp., Armonk, USA) and table by Microsoft Excel (Microsoft Corporation, Redmond, USA). Risk factors for recurrence and complications were also recorded for analysis.

## Results

In the current study, 130 patients who had all had buccal mucosal graft (BMG) urethroplasty for anterior stricture were examined. These patients' average ages ranged from 12 to 71 years, with a mean of 41.4 years. There were 20 males afflicted by the overall recurring stricture incidence, which was found to be 15.4%. Diverse presenting management techniques were used to treat the recurrence in these patients, including clean urethral dilation and direct vision internal urethrotomy (DVIU) (n = 20), intermittent self-catheterization (n = 10), long-term suprapubic catheterization (n = 4), urethral perineal urethrostomy (n = 5), and redo urethroplasty (n = 3). Twenty patients (15.4%) had postoperative complications, the majority of which occurred after penile urethroplasty: urinary fistula (n=4, 3%), graft contracture (n=3, 2.3%), graft failure (n=5, 3.8%), UTI (n=4, 3%), wound infection (n=3, 2.3%), and secondary bleeding from the mouth (n=1, 0.7%)(Table 1). Two cases of spontaneous healing of the urinary fistula were noted, two cases were corrected, and five cases needed revision surgery, three of which were carried out during the second stage of graft. During follow-up, Acute urine retention (AUR) was recorded in 23% of patients (n=30), post-micturition dribbling (PMD) in 14% of patients (n=18), and erectile dysfunction (ED) in 11% of patients (n=14). While all ED patients had penile artery and venous Doppler ultrasonography for more than a year, all cases of PMD were treated conservatively. An oral phosphodiesterase type 5 (PDE5) inhibitor was used to treat ED once it was discovered to be non-organic. After undergoing penile urethroplasty, one patient with severe Balanitis Xerotica Obliterans (BXO)** **and many prior reconstructions reported an unsatisfactory aesthetic result (3.8%), but the functional outcome was deemed successful. Age at surgery, aetiology, stricture length, and place were found to be significant predictors of stricture recurrence in a univariate analysis.

**Table 1:** Post-operative complication mainly after penile urethroplasty

Complication	N (%)
Urinary fistula	4 (3)
Graft contracture	3 (2.3)
Graft failure	5 (3.8)
Urinary tract infection	4 (3)
Wound infection	3 (2.3)
Secondary bleeding from mouth	1 (0.7)

In contrast to individuals 30 years of age, who only experienced a recurrence in two cases (7.1%), failure risk was seen in patients aged 31-50 years (12.1%, n=8, P<0.05) and >50 years (27.7%, n=10, P<0.05). In addition, BXO was observed to predict treatment failure in 26 individuals with penile stricture associated with this disease, with stricture recurrence occurring in 30.7% (n=8，P<0.05) of these patients. Stricture length was divided into three categories: 4 cm (n=70), 4.1-8 cm (n=35), and >8 cm (n=25). Whereas failure rates were 20.0% (n=7, P<0.05) and 44.0% (n=11, P<0.05), respectively. According to the location of the disease, statistically significant stricture recurrence was shown: although only 4.4% (n=3) of 68 patients with bulbar urethral stricture presented with recurrence during follow-up, 27.4% (n=17, P<0.05) of 62 patients noted peno-bulbar stricture. The penile and peno-bulbar location was the sole significant independent variable for re-stricture on multivariate analysis.

Additionally, it was discovered that there was a greater prevalence of problems at the site of the penile stricture: 17 complications were recorded, with 13 instances following penile urethroplasty, three cases of wound-side infection, and one case of secondary infection in cases of bulbar urethral stricture. Only the stricture length was shown to be linked to a greater incidence in the univariate/multivariate analysis, while PMD and ED were not found to be related to age, aetiology, or stricture site (Table 2).

**Table 2:** Risk factor for stricture recurrence and side effect after penile and bulbar BMG urethroplasty ^*^P<0.05 value indicates statistically significant risk factors for stricture recurrence. BMG: buccal mucosal graft, PMD: post-micturition dribbling, ED: erectile dysfunction; TURP: transurethral resection of the prostate.

Total N (%)	N 130 (%)	Mean (SD)	Restricture N 20 (%)	PMD, N 18 (%)	ED>12 months, N 14 (%)	Comesis N 1 (%)	T-test	Chi-Square	P-Value
Age, years [N]									
≤30	28 ( 21.54)	2(1.41)	2(7.1)	4(14.2)	4 (14.2)	0	-	-	-
31–50	66(50.77)	4(2.54)	8(12.1) *	9 (13.6)	7(10.6)	1(4.5)	2.3	6.1	<0.05
>50	36(27.69)	3.4(1.94)	10(27.7) *	5(13.8)	3 (8.3)	0	3.5	8.4	<0.05
Aetiology									
Idiopathic	44(33.85)	4(2.56)	4(9.0)	7(15.9)	3(6.8)	0	-	-	-
BXO	26 (20)	8(5.51)	8(30.7) *	1(3.8)	2(7.7)	1(3.8)	4.2	9.2	<0.05
Hypospadias	23(17.69)	3(2.65)	3(13.0)	3(13.0)	2(8.7)	0	-	-	-
Iatrogenic	14(10.77)	3(1.73)	3(21.4)	2(14.2)	3(21.0)	0	-	-	-
Infection	6(4.62)	0(0)	0	2(33.3)	0	0	-	-	-
Trauma	10 (7.69)	1(1.58)	1 (10.0)	2(20.0)	2(20.0)	0	-	-	-
TURP	7(5.38)	1(1.46)	1 (14. 2)	1(14.2)	2(28.5)	0	-	-	-
Stricture length [cm]									
≤4	70 (53.85)	2(1.55)	2 (2.6)	3(4.2)	4(5.7)	1(1.4)	-	-	-
4.1–8	35(26.92)	7(0)	7(20.0) *	7(20.0)	6(17.4)	0	3.0	7.6	<0.05
>8	25(19.23)	11(0)	11(44.0)*	8(32.0)	4(16.0)	0	4.8	10.1	<0.05
Stricture site									
Bulbar	68(52.31)	3(0.77)	3(4.4)	10(14.7)	7(10.2)	0	-	-	-
Penile /Peno-bulbar	62(47.69)	17(4.78)	17(27.4)*	8(12.9)	7(11.2)	1(1.6)	3.2	8.9	<0.05

## Discussion

In 1941, Humby and Higgins published the first description of the use of buccal mucosal graft (BMG) for urethral repair [6]. Due to its simplicity in harvesting, lack of hair, favourable surgical handling properties, compatibility with a moist environment, and early development and graft survival, it has now come to be preferred over other types of grafts. In fact, during the 1990s, it grew in popularity. The most effective method for treating urethral stricture is still open urethroplasty. In 1993, El-Kasaby et al. published the first case study of buccal mucosal urethroplasty for the treatment of penile and bulbar urethral strictures [7]. The dorsal onlay graft urethroplasty performed on 12 patients by Barbagli et al. in 1996 demonstrated success in all cases after that [2]. Similar outcomes were also reported by Morey and McAninch in 13 patients who had anterior urethral stricture treatment with ventral onlay BMG urethroplasty [8]. In a recent study, Barbagli found that dorsal onlay bulbar urethroplasty using BMG had an 80.2% long-term success rate. Variations of this method, according to Kulkarni, have success rates as high as 92%. In a multicenter retrospective research performed in 2008 by Barbagli and Kulkarni, 359 patients who had bulbar and penile augmentation urethroplasty utilising penile skin or oral mucosal graft, respectively, demonstrated long-term success rates of 73.8% and 74.1% [9-10]. With a success rate of up to 87%, the Asopa technique reported the same procedure for dorsal inlay-free graft urethroplasty [11]. The disparity in success rates, however, may be attributable to variations in geographic location, sample, and BMG urethroplasty types. Comparable results between dorsal onlay and inlay methods have been shown by randomised studies and systematic reviews [12-13]. In this article, we discuss our experiences treating penile and bulbar urethral strictures with graft augmentation urethroplasty. According to the literature, up to 90% of urethral strictures occur in the anterior urethra, and up to two-thirds of up to 75% occur in the bulbar urethra [14-16], the majority of patients, 68 (52.31%), presented with a stricture at the bulbar site. Our dorsal onlay BMG urethroplasty used the Barbagli procedure to treat bulbar urethral stricture. If a sufficient urethral plate was available, the Asopa procedure was the recommended method for treating penile urethral stricture; otherwise, a total urethral graft replacement with the removal of diseased tissue was carried out. Our study reported penile urethral stricture had a success rate of 72.6% and bulbar urethral stricture of 95.6%. However, the penile location did not respond to either univariate or multivariate analysis 17 (24%, P<0.05) failure rate with predictor for restricture. Mohamed et al. (91.1%) [17] and Tavakkoli Tabassi et al. (93.3%) [18] reported comparable success rates for the bulbar urethra, whereas Spilotros et al. (81%) [19] and Barbagli et al. (80%) [20] reported lower success rates.

In a recent retrospective research, Barbagli showed that penile and bulbar urethroplasty had similar outcomes [9]. For a normal penis, penile urethroplasty is a straightforward treatment, but it carries a greater chance of failure in men who come with severe BXO and failed hypospadias. The study shows that the penile urethroplasties were done in complicated strictures on 36 (58.0%) patients who had a recurrent illness, 26 (41.9%) of whom had previously had hypospadias surgery, and 22 (35.4%) of whom had BXO. Our study also looked at the aetiology (BXO = P<0.05), the duration of the stricture, and several prior therapies.

Patel et al. [21] reported on 79 individuals who had penile and bulbar urethral stricture as a result of BXO in a recent retrospective study. They reported success rates that were 76% for two-stage BMG urethroplasty and 75% for one stage. An increased likelihood of recurrence is connected with variables such long segment stricture 5 cm, as well as lichen sclerous, infectious, and iatrogenic aetiology, according to Kinnarid et al.'s [22] study on a comprehensive retrospective assessment of 604 cases. With a stricture length of less than 5 cm, Awad et al.'s [1] study of 60 patients revealed a success rate of 100%; however, with a stricture length of more than 5 cm, the failure rate was 13%, and the restructuring was discovered at the proximal and distal end of the graft anastomosis. These findings were in line with systemic review studies by Yalcinkaya and Meek [5,23], which revealed a significant difference in the failure rate of 12.4% for strictures that were less than 5 cm in length, but a failure rate of 16.6% was seen for strictures that were larger than 5 cm. Similar results were found in our research, where only 2.6% of patients with strictures under 4 cm did not experience recurrence during follow-up, compared to recurrence of 20.0% (P<0.05) and 44.0% (P<0.05) for strictures between 4.1 to 8 cm and >8 cm.

The results of the current study show that patients under the age of 30 who had stricture therapy had better outcomes, with a recurrence rate of 7.1% as opposed to individuals between the ages of 31 and 50 years (12.1%, P<0.05) and above 50 (27.7%, P<0.05). Similar findings were seen in Awad et al.'s [1] research, where patients under the age of 20 had a recurrence rate of 3.7% compared to those aged 20 to 40 years, who had 40.7%, 44 to 60 years, who had 44.4%, and 11.1% over the age of 60 years. For patients who had bulbar urethroplasty, Barbagli reported a success rate of 89.9% for those under 65 and a success rate of 100% for those over 65 [24].

In this study, we found that complications occurred at a rate of 15.4%, primarily in the group receiving penile urethroplasty (9.2% vs. 6.10%). Urinary fistula, graft contraction, graft failure, and urinary tract infections (UTI) accompanying wound infection were the most prevalent side effects. According to Awad et. al [1], urinary tract infections (23.3%) and superficial wound infections (20%) were the most common post-operative sequelae. Additionally, in the research by Spilotros et al. [19], urinary fistula (3.1%), graft contracture (3.1%), and graft failure (3.1%) were the most often seen problems. The main postoperative consequence, according to Javali et al., was wound infection [4]. Patients with problems such as fistula (5.7%), wound infection (1.9%), and meatal stenosis (2.9%) were identified by Mohamed et al. [17]. The revision rate was 32.3% (n = 42), which included a fresh urethroplasty operation for recurrent stricture. Mori et al. [25] found that in 78 patients who had a multistage repair for challenging anterior urethral strictures, there were 10.3% of revisions and a 19.2% total complication rate.

The study on Buccal Mucosal Graft (BMG) urethroplasty for anterior urethral stricture has several notable limitations that impact its validity and generalizability. Firstly, its retrospective design introduces potential biases due to the non-randomized selection of patients, making it difficult to establish causal relationships. Additionally, the data are sourced from a single center in Nepal, which raises concerns about the applicability of the findings to broader populations with different healthcare contexts. The absence of a control group further complicates the ability to compare outcomes effectively against other treatment modalities. Moreover, the 48-month follow-up period may not capture long-term complications or the durability of outcomes, and variations in surgical technique may introduce additional variability in results. Other limitations include potential patient selection bias, a small sample size that reduces statistical power, and reliance on subjective outcome measures, which could skew findings.

## Conclusions

The research undertaken in Nepal provides pivotal insights into the outcomes of Buccal Mucosal Graft (BMG) urethroplasty for anterior urethral stricture in males. With a substantial success rate of 84.6% over a 48-month follow-up, the procedure stands as a promising intervention for such conditions. Notably, the research highlighted specific factors, such as the penile location of the stricture, which appeared as a significant independent predictor for re-stricture. Moreover, postoperative complications were observed in 15.4% of the patients for stricture recurrence. The study's findings also reflect the advancements in surgical techniques and healthcare delivery in resource-limited settings like Nepal, emphasizing the potential of such interventions in similar environments. The favorable outcomes of BMG urethroplasty, as evidenced by this study, reinforce its viability as a primary approach for treating anterior urethral strictures. Further research and continuous monitoring can further refine the technique and optimize patient outcomes.
